# The Diverse Roles of microRNAs at the Host–Virus Interface

**DOI:** 10.3390/v10080440

**Published:** 2018-08-19

**Authors:** Annie Bernier, Selena M. Sagan

**Affiliations:** 1 Department of Microbiology & Immunology, McGill University, Montréal, QC H3G 1Y6, Canada; Annie.bernier@mail.mcgill.ca; 2 Department of Biochemistry, McGill University, Montréal, QC H3G 1Y6, Canada

**Keywords:** microRNAs, Herpesviruses, polyomaviruses, retroviruses, pestiviruses, hepaciviruses, latency, immune evasion, viral RNA accumulation

## Abstract

MicroRNAs (miRNAs) are small, non-coding RNAs that regulate gene expression at the post-transcriptional level. Through this activity, they are implicated in almost every cellular process investigated to date. Hence, it is not surprising that miRNAs play diverse roles in regulation of viral infections and antiviral responses. Diverse families of DNA and RNA viruses have been shown to take advantage of cellular miRNAs or produce virally encoded miRNAs that alter host or viral gene expression. MiRNA-mediated changes in gene expression have been demonstrated to modulate viral replication, antiviral immune responses, viral latency, and pathogenesis. Interestingly, viruses mediate both canonical and non-canonical interactions with miRNAs to downregulate specific targets or to promote viral genome stability, translation, and/or RNA accumulation. In this review, we focus on recent findings elucidating several key mechanisms employed by diverse virus families, with a focus on miRNAs at the host–virus interface during herpesvirus, polyomavirus, retroviruses, pestivirus, and hepacivirus infections.

## 1. Introduction

MicroRNAs (miRNAs) are small non-coding RNA molecules, typically 21 to 25 nucleotides (nt) in length, that are highly evolutionarily conserved, developmentally regulated and are expressed in a tissue-specific manner. Since their description in the early 1990s, miRNAs have been found in over 200 species, with over 2000 human miRNAs and more than 24,000 entries in the Sanger database [1,2]. MiRNAs are typically transcribed by RNA polymerase II as long, highly structured primary miRNA transcripts (pri-miRNAs), often found in the introns of protein-coding genes [3,4]. These pri-miRNAs are then processed into short, ~70 nt hairpin-shaped precursor miRNAs (pre-miRNAs) by Drosha, a nuclear RNase III enzyme [5]. In the cytoplasm, the pre-miRNAs are further processed by the RNase III enzyme, Dicer, into mature ~22-nt miRNA duplexes [6,7,8]. Dicer interacts with the transactivation response RNA-binding protein (TRBP) and the protein activator of PKR (PACT), two dsRNA-binding proteins that play roles in miRNA processing efficiency and specificity [9]. Following Dicer cleavage, the mature miRNA is loaded into an Argonaute (Ago) protein, an essential component of the RNA-induced silencing complex (RISC) [10]. The Ago protein unwinds the mature miRNA duplex and uses one strand, known as the guide strand, to target mRNAs in a sequence-specific manner. While miRNAs usually bind to the 3′ untranslated region (UTR) of their target mRNAs, they have also been reported to target the 5′ UTR and coding sequences of mRNAs [11,12,13]. Although both strands of a duplex can serve as the guide strand, strand selection is determined by the duplex stability at the 5′ end of each miRNA arm, and highly abundant miRNAs typically originate more frequently from the 5p strand than the 3p strand [14,15]. Pairing interactions between miRNAs and their target RNAs primarily involve nucleotides 2–8 of the miRNA, which is referred to as the “seed sequence” [16]. However, additional pairing to the 3′ region of the miRNA can compensate for mismatches in the seed region [17]. Perfect complementarity or extensive centered pairing results in target mRNA cleavage, whereas imperfect base-pairing typically results in translational inhibition and/or accelerated deadenylation, culminating in repression of target gene expression [18,19]. In mammals, miRNAs are typically imperfectly complementary to their targets, whereas in plants and insects they may have perfect complementarity [20]. Since miRNAs can bind to their targets with imperfect complementarity, a single miRNA can target over 100 genes, and a single gene may be regulated by multiple miRNAs [21,22]. In fact, it is predicted that over two-thirds of all human genes are targeted by miRNAs [23]. Thus, it is not surprising that miRNAs are implicated in many cellular processes, including cell proliferation, differentiation, apoptosis, metabolism, host immunity, and viral infections [24,25,26,27].

MiRNA-mediated regulation of viral infection has been described in a wide variety of hosts and across both DNA and RNA virus families. Several types of interactions have been observed, including: cellular miRNAs directly targeting host or viral transcripts; evasion of cellular miRNAs; broad impairment of the miRNA pathway; and even virally encoded miRNAs that regulate host or viral gene expression. Such interactions have been described to play crucial roles in the regulation of viral replication, maintenance of latency and/or reactivation, immune evasion, and cell transformation. Herein, we highlight recent studies elucidating the role of miRNAs at the host–virus interface, with a focus on several key canonical and non-canonical miRNA interactions that contribute to viral infection and pathogenesis in a select group of well characterized DNA and RNA viruses.

## 2. Herpesviruses

Herpesviruses are large dsDNA viruses that can establish lifelong infections and cycle between lytic (productive) and latent (non-productive) replication [28]. This family is divided into three subfamilies with general differences in the cell types involved: the *Alphaherpesvirinae* latently infect neurons; *Betaherpesvirinae* are found in the monocyte lineage; and *Gammaherpesvirinae* infect lymphocytes [29]. Clinical manifestations of herpesvirus infections can range from skin and mucosal lesions to severe malignancies and deadly encephalitis. The most well-studied herpesviruses include five common human pathogens: herpes simplex viruses type 1 and 2 (HSV-1 and HSV-2), human cytomegalovirus (HCMV), Epstein-Barr virus (EBV), and human herpesvirus 8 (HHV-8), also known as Kaposi’s sarcoma-associated herpesvirus (KSHV) [30]. The role of miRNAs in the pathogenesis of herpesvirus infection has been extensively studied and provides a good overview of both canonical and non-canonical interactions with the miRNA pathway during viral infection (reviewed in [31]).

### 2.1. Cellular miRNAs in Herpesvirus Infection

#### 2.1.1. MiRNAs Implicated in Latency Maintenance

Herpesviruses are characterized by their ability to carry out lytic replication or establish latency, and several cellular miRNAs have been implicated in this process. HSV-1 latency is promoted by two cellular miRNAs, miR-101 and miR-138 (Table 1). Expression of the HSV-1 immediate early protein ICP4, a key transactivator of early and late viral genes, induces the expression of miR-101 by directly binding to and activating its promoter [32,33]. In turn, miR-101 directly downregulates expression of the mitochondrial ATP synthase subunit beta (ATP5B), a protein known to promote HSV-1 replication [34]. ATP5B depletion was shown to block HSV-1 DNA packaging and capsid maturation, presumably by limiting the energy available to complete the viral life cycle [35]. MiR-101 also downregulates the RNA-binding protein G-rich sequence factor 1 (GRSF1), whose binding to HSV-1 p40 mRNA typically enhances its expression, facilitating viral replication [33]. Therefore, induction of miR-101 expression during HSV-1 infection downregulates both ATP5B and GRSF1 expression, consequently attenuating viral replication and preventing lytic cell death. In addition, the neuron-specific miR-138 directly downregulates the expression of another viral transactivator of lytic gene expression, ICP0. Thus, both miR-101 and miR-138 have been demonstrated to inhibit lytic gene expression and promote viral latency during HSV-1 infection [36].

Similarly, HCMV is a ubiquitous pathogen that is able to establish latent infection upon resolution of acute infection [37]. Reactivation of the HCMV lytic cycle during times of immunological stress can result in severe disease and mortality [38]. The HCMV immediate early transcript, UL112, one of the two early proteins known to initiate viral reactivation, is the direct target of three human miR-200 family members: miR-200b, miR-200c, and miR-429 (Table 1) [39]. This cluster of miRNAs is highly expressed in undifferentiated cells such as monocytes, but lost during differentiation, and this is thought to act as a switch for viral reactivation [39].

Following primary infection, EBV can also establish a latent infection in the nucleus of memory B cells. Although usually benign, EBV infections are known for their ability to transform infected cells and have been associated with many cancer types, including several lymphomas [40]. Similarly to the suggested role of the miR-200 family in HCMV reactivation, miRNAs from the miR-200 cluster have also been shown to induce EBV reactivation (Table 1) [41]. Specifically, miR-200b and miR-429 directly downregulate the expression of the two host proteins, ZEB1 and ZEB2, that repress the transcription of the EBV immediate-early transcription factor, BZFL1 [42]. Thus, miR-200b and miR-429 indirectly allow expression of BZFL1, which induces early lytic gene expression and binds to the origin of replication of the EBV genome promoting viral replication. In contrast, miRNAs are also implicated in EBV latency and expression of the viral protein EBNA1 transactivates the expression of let-7a primary transcripts during infection [43]. EBNA1 is expressed during both lytic and latent infections and is required for EBV replication as well as segregation of episomal genomes during latency [44,45]. EBNA1-mediated upregulation of let-7a results in direct downregulation of Dicer gene expression, thereby decreasing overall cellular miRNA levels and reinforcing latency [43]. EBNA1 expression also results in a significant decrease in BZFL1 expression without modulating miR-200b or miR-429 levels, suggesting that EBNA1-mediated upregulation of let-7a may reinforce latency independently of ZEB1 and ZEB2 levels.

Finally, although less is known regarding the role of cellular miRNAs in KSHV latency, miR-320d, miR-498 and miR-1258 have all been shown to bind to the 3′ UTR of the KSHV replication and transcription activator (RTA) transcript (Table 1). Downregulation of this important reactivation factor results in a repression of KSHV reactivation [46,47]. Thus, by targeting both host and viral transcripts (Table 1), cellular miRNAs appear to be major regulators of latency and/or viral reactivation during infection with several human herpesviruses.

#### 2.1.2. MiRNAs Implicated in Immune Evasion

Whether latency is an immune evasion strategy or a form of tolerance is subject to some debate (reviewed in [67]). The restriction of gene expression during latency significantly reduces the abundance of viral antigens available for presentation to immune cells. However, herpesviruses have also evolved distinct strategies to more directly inhibit host immune responses using miRNAs. For example, miR-23a is upregulated during HSV-1 infection, and downregulates interferon regulatory factor 1 (IRF1) gene expression, impairing the interferon pathway and leading to innate immune evasion [51]. This also results in the downregulation of the antiviral gene RSAD2, known to limit HSV-1 replication [52]. MiR-649 further promotes HSV-1 replication by directly targeting the mucosa associated lymphoid tissue lymphoma translocation gene 1 (MALT1) [53]. Downregulation of MALT1 results in evasion of both innate and adaptive immune responses through inhibition of the NF-κB pathway [54]. Of note, miR-649 levels were shown to be downregulated following HSV-1 infection in HeLa cells, and hence its downregulation may play a role in limiting HSV-1 replication through a negative feedback loop.

Similarly, during KSHV infection, upregulation of miR-132 results in repression of interferon-stimulated genes (ISGs) by targeting an important transcriptional coactivator [55]. A similar function for miR-132 has also been described in HSV-1 and HCMV infection [55]. Thus, as these examples illustrate, cellular miRNA-directed immune evasion during herpesvirus infection mainly occurs through downregulation of key signaling proteins in antiviral immune pathways.

#### 2.1.3. MiRNAs Implicated in Cell Cycle Control and Tumorigenesis

Cellular miRNAs are also key players in the regulation of herpesvirus-induced tumors. For example, upregulation of miR-190 expression during EBV infection directly results in downregulation of TP53INP1, leading to enhanced cell survival by inhibiting apoptosis and cell cycle arrest [58]. Furthermore, upregulation of miR-424 and miR-127 during EBV infection promotes lymphomagenesis by downregulating the tumor suppressor ubiquitin ligase SIAH1 and blocking B-cell differentiation, respectively [59,60,68]. Interestingly, the viral protein EBNA1, previously discussed for its role in promotion of the latency by inducing let-7a expression, also leads to upregulation of miR-127 [60].

Like EBV, KSHV is the etiologic cause of tumorigenesis, including Kaposi’s sarcoma, a tumor of lymphatic endothelial lineage [69]. One mechanism by which KSHV is known to induce cancer involves interleukin-6 (IL-6), a cytokine that promotes cell growth, angiogenesis and lymphoma formation. KSHV infection induces tumorigenesis through upregulation of host interleukin-6 (IL-6), a cytokine that promotes cell growth, angiogenesis, and lymphoma formation [70,71]. In addition, KSHV also encodes a viral mimic of human IL-6 (vIL-6). However, cellular miR-608 and miR-1293, respectively, can downregulate human IL-6 and vIL-6 expression directly through binding to sequences in their open reading frames (ORFs) [72]. On the other hand, the viral ORF57 protein can compete with these miRNAs for binding to these sequences on the IL-6 and vIL-6 mRNAs [73]. ORF57 binding thus masks these miRNA sites, and subsequently results in stabilization of these transcripts, promoting IL-6 and vIL-6 gene expression. Furthermore, KSHV infection results in upregulation of miR-21 and miR-31, which directly target two tumor suppressors linked to neoplastic transformation, cell migration and angiogenesis [61,62,63,64]. Upregulation of miR-146a is also implicated in KSHV-infected cell migration and spread by directly targeting the chemokine receptor CXCR4, which promotes the premature release of endothelial cell progenitors into the blood stream [65]. Of note, both miR-21 and miR-146 have been detected inside KSHV virions and were reported to retain their biological functionality during de novo infections [74]. Evidence suggests that these so-called “virional” miRNAs are selectively packaged inside virions during encapsidation or envelopment, but it is still unclear if they directly contribute to infection or pathogenesis in vivo. However, similar virional miRNAs have been reported in other viral infections, including HCMV and HIV-1, suggesting that this may be a common strategy used to control gene expression early in infection [75,76].

Thus, it is clear that herpesviruses modulate cellular miRNA expression in order to regulate latency, evade antiviral immune responses and promote tumorigenesis. The identification of virional miRNAs provides an additional layer of complexity and further research will be required to elucidate whether this provides a distinct advantage to the virus during de novo infection.

### 2.2. Herpesvirus-Encoded miRNAs

An important characteristic of herpesviruses that distinguishes them from many other virus families is their ability to express several virally encoded miRNAs [77,78]. Since the discovery of the first viral miRNA of EBV in 2004, more than 500 viral miRNAs have been identified across several diverse virus families [2,79,80]. To date, 8 of the 9 human herpesviruses have been shown to encode at least one miRNA [81]. These miRNAs have been reported to target both cellular and viral transcripts and play significant roles in regulating latency and evading host immune responses, both of which will be described in more detail below.

#### 2.2.1. Viral miRNAs with Cellular Targets

Although most identified targets of the 27 mature HSV-1 miRNAs are viral transcripts, recent research has described roles for these viral miRNAs in targeting cellular transcripts to promote immune evasion, viral replication, cell proliferation, and pathogenesis [82,83]. One such example is the targeting of PIGT by miR-H8. PIGT is an important component of the glycosylphosphatidylinositol anchoring pathway that allows proteins to be presented on the cell surface. MiR-H8 represses PIGT expression resulting in a reduction in presentation of many immune-related proteins, including NK-cell ligands and the viral restriction factor, tetherin [83]. Thus, by targeting PIGT, miR-H8 efficiently counteracts the host immune response at several key points. In addition, the HSV-1 miR-H1 directly targets the ubiquitin protein ligase 3 component, Ubr1, a crucial component of the ubiquitin-proteasome system [84,85]. MiR-H1-mediated downregulation of the ubiquitin-proteosome system results in accumulation of neurodegenerative-associated protein fragments, and thus may play a role in HSV-1 pathogenesis [84].

In contrast to HSV-1, most of the 21 mature HCMV-encoded miRNAs are thought to target cellular transcripts, with well-defined roles in immune evasion from NK cell-mediated killing [31]. HCMV-miR-UL112 targets the major histocompatibility complex (MHC) class-I related chain B (MICB), a NKG2D ligand, to reduce NK-mediated killing [86]. The viral miRNA binding site overlaps with that of the cellular miR-367a, which suggests that HCMV may have evolved to prevent target site mutations by targeting highly conserved sequences [87]. Of note, EBV BART-2-5p and KSHV K-12-1 were also reported to target MICB to promote immune evasion [88]. Two other viral miRNAs, miR-US25-3p and miR-UL148D, also contribute to evasion from NK cell recognition by targeting tissue inhibitors of metalloprotease 3 (TIMP3) and the chemokine receptor CCL5, respectively, resulting in increased shedding of MHC class-I related chain A (MICA) and inhibition of NK cell proliferation and activation [89,90,91]. Additional roles for HCMV miRNAs have been reported in cell cycle control, regulation of latent and lytic infection, and in vesicle trafficking to support virion assembly [31,92].

EBV encodes at least 44 mature miRNAs with described roles in immune evasion, inhibition of apoptosis, cell transformation, and maintenance of latency [31,93,94,95]. As examples, miR-BHRF1-3 targets CXCLL11, a T-cell attracting chemokine [96]; miR-BART2-5p (as discussed above) targets the NK cell ligand MICB [88]; and miR-BART6-3p downregulates the expression of the antiviral RNA helicase, retinoic acid-inducible gene I (RIG-I) [97]; all contributing to EBV-mediated evasion of host innate immune responses. On the other hand, one of the most well characterized targets of EBV miRNAs is the pro-apoptotic protein PUMA, which is downregulated by miR-BART-5p to avoid apoptosis of EBV-infected cells [98]. Targeting of at least three other pro-apoptotic genes as well as multiple tumor suppressors by several EBV miRNAs has also been reported, suggesting they contribute to EBV-induced tumorigenesis and cell transformation [31,99]. Interestingly, EBV miR-BART6-5p, may also play a critical role in maintenance of latency by directly targeting the human Dicer transcript, resulting in repression of miRNA biogenesis and reinforcing the previously discussed strategy of let-7-mediated repression of miRNA expression during EBV infection [43,95]. Since Dicer is also required for viral miRNA processing, this suggests a negative feedback loop may tightly regulate miRNA levels. The downregulation of Dicer also results in decreased expression of EBV lytic transactivators (Zta and Rta) and EBNA2, which further reinforce latency. Thus, EBV encoded miRNAs act on host transcripts to promote both immune evasion and viral latency [100,101].

Finally, KSHV encodes at least 25 mature miRNAs that are implicated in immune evasion and tumorigenesis. Three KSHV miRNAs (miR-K12-1, -K12-6 and -K12-7) have been demonstrated to target MCP-1-induced protein 1 (MCPIP1) transcripts, a protein implicated in suppression of miRNA biosynthesis (through cleavage of pre-miRNAs) and negative regulator of inflammation through cleavage of IL-6 mRNA [102,103,104]. In addition, miR-K12-3 and miR-K12-7 downregulate the expression of C/EBPβ, a translational repressor of IL-6 and IL-10 [105]. Thus, combined with previously discussed effects of viral ORF57 competition for cellular miRNA target sites on expression of vIL-6 and IL-6 mRNAs, this leads to a further upregulation of IL-6 expression, further promoting cell growth, angiogenesis and lymphoma formation. These three mechanisms demonstrate how viral products, including several miRNAs, can have both cooperative and redundant functions during viral infection.

In addition to de novo targeting, three KSHV miRNAs share seed sequences with cellular miRNAs, making them functional orthologs able to tap into established cellular miRNA target networks [106,107,108,109]. Through this activity, miR-K12-10 (miR-142-3p ortholog) inhibits the TGF-β pathway to promote cell survival [110]; miR-K12-3 (miR-23 ortholog) inhibits caspases 3 and 7 to inhibit apoptosis [109]; and miR-K12-11 (miR-155 ortholog) represses several signaling pathway components of the interferon response and promotes cell survival [111,112,113,114,115]. Thus, KSHV modulates cellular gene expression through de novo targeting, but also uses miRNA mimicry to tap into established cellular miRNA target networks. Through these activities, KSHV miRNAs promote immune evasion, cell survival, and tumorigenesis.

#### 2.2.2. Viral miRNAs Regulating Viral Transcripts

While cellular miRNAs play crucial roles in maintaining latency, several virally encoded miRNAs have also been shown to promote latency through targeting viral transcripts. HSV-1 miR-H6 represses expression of the viral protein ICP4, an immediate early gene that normally promotes the lytic cycle through transcriptional activation of early and late genes as well as through downregulation of LAT expression [116]. Moreover, similarly to miR-138, miR-H12 represses ICP0 expression, a viral transactivator of early gene expression [117,118]. Two other HSV-1 miRNAs have also been shown to promote latency by targeting a lytic neurovirulence factor (ICP34.5), and all four of these HSV-1 miRNAs implicated in viral latency were found to be upregulated during the latent cycle [117,119,120,121]. In contrast to HSV-1, very few HCMV or EBV miRNAs have been reported to target viral transcripts. However, HCMV miR-UL112-1 was shown to downregulate the major immediate early transactivator, IE72, resulting in a reduction of viral replication and promotion of latency [122]. During EBV infection, miR-BART-5p, miR-BART15 and miR-BART17-5p all downregulate expression of the viral latency-associated membrane protein, LMP1 [123]; while miR-BART22 targets LMP2A [124]. Since LMP1 and LMP2A are known viral antigens that can induce potent cytotoxic CD4^+^ and CD8^+^ T cell responses and NF-κB signaling, their downregulation by EBV miRNAs is predicted to play a role in both latency maintenance and immune evasion [123,125,126].

Like HSV-1, KSHV miRNAs that target viral transcripts have well-described roles in the maintaining latency (reviewed in [127]). This role is in accordance with their location in the viral genome as all KSHV miRNAs are encoded within the latency-associated region [128]. MiR-K12-7 and miR-K12-9 both directly downregulate the viral protein RTA, a protein essential for initiation of lytic replication [129,130]. In addition, several other KSHV miRNAs indirectly inhibit RTA expression, by silencing the RTA promoter or repressing known RTA activators [131,132]. Taken together, the majority of herpesvirus miRNAs that target viral transcripts appear to play important roles in latency maintenance. However, although viral targets have not been confirmed for the vast majority of herpesvirus miRNAs, their differential expression during latent and lytic cycles suggest additional as of yet unidentified roles for these miRNAs in regulation of latency [133].

In summary, while the targets of herpesvirus-encoded miRNAs are still being elucidated, it is clear that these miRNAs play a major role in regulating gene expression in infected cells. Through their cellular targets, herpesvirus miRNAs promote immune evasion, cell survival and tumorigenesis; while the viral targets appear to be crucial for latency maintenance. The variety of diverse mechanisms utilized by herpesviruses, including broad regulation of the miRNA pathway, virally encoded miRNAs and miRNA mimicry, is likely a reflection of the long co-evolution of herpesviruses with the miRNA pathway.

## 3. Polyomaviruses

**Figure 1:**
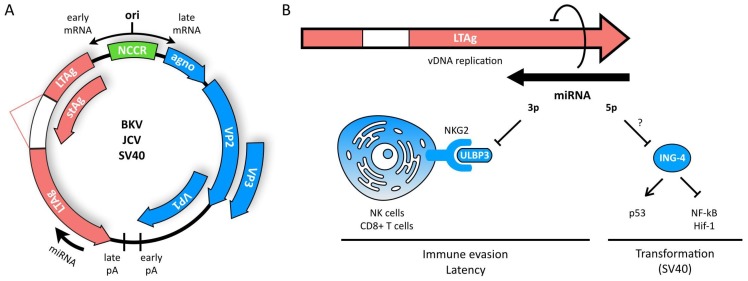
Betapolyomaviruses encode a miRNA in the LTAg region of the genome. (**A**) Genomic organization of the beta polyomaviruses BKV, JCV and SV40. The genome is divided into an early and late region encoded on opposite strands and separated by a non-coding control region (NCCR). The late strand of the genome encodes three capsid proteins (VP1-3) and two mature miRNAs originating from the same pre-miRNA located at the 3′ end and antisense to the large tumor antigen (LTAg) gene. The polyadenylation sites (pA) are involved in the early-to-late switch. (**B**) Perfect complementarity of the miRNA to the early viral LTAg mRNA results in direct cleavage and inhibition of LTAg expression to promote latency. The 3p arm of the miRNA directly downregulates expression of the stress-induced ligand ULBP3, contributing to immune evasion. The SV40-encoded miRNA 5p arm mimics hsa-miR-423 and is therefore suggested to contribute to cell transformation by downregulating the tumor suppressor ING-4.

Polyomaviruses (PyVs) are non-enveloped viruses with a circular dsDNA genome of ~5 kb [134,135]. The PyV family includes >70 species classified in 4 genera that infect a wide range of hosts including humans, primates, birds, rodents, and cattle [136]. Although most infections by PyVs are asymptomatic, PyV infection increases the overall incidence of tumor formation (reviewed in [137]). The PyV family shares a similar genomic organization in which the genome is divided into an early and late region encoded on opposite strands (Figure 1A) [138]. The betapolyomaviruses BK virus (BKV), JC virus (JCV), and simian virus 40 (SV40), encode two mature miRNAs originating from a single pre-miRNA found at the 3′ end, antisense to the large tumor antigen (LTAg) gene [135]. The miRNAs encoded by BKV (BKV-miR-B1-5p and 3p) and JCV (JCV-miR-J1-5p and 3p) have a very high sequence similarity, while the 5p and 3p arms of the SV40 miRNA (SV40-miR-S1) have only 50 and 75% sequence identity to the BKV and JCV miRNAs, respectively [139].

The 5p BKV and JCV-encoded miRNAs are more abundantly expressed than the 3p miRNAs, and all PyV miRNAs show expression late during infection [139,140,141]. This observation is consistent with PyV miRNAs being encoded on the late strand. Each miRNA is perfectly complementary to a region of the early viral LTAg mRNA and can therefore direct cleavage, resulting in inhibition of LTAg expression [135,139,142,143]. This downregulation of LTAg results in impairment of viral DNA replication and reduced recognition of PyV-infected cells by cytotoxic T lymphocytes [140]. Additionally, the 3p BKV and JCV miRNAs downregulate stress-induced cell surface markers, reducing NK and CD8^+^ T cell mediated killing of PyV-infected cells and contributing to immune evasion (Figure 1B) [144,145]. PyV-encoded miRNAs are therefore considered to play an important autoregulatory role in limiting viral replication, as well as in suppression of the immune response to infection [140]. These mechanisms may thus control the establishment of viral latency and/or persistence. However, recent data suggests that murine PyV miRNAs do not only play a role in limiting viral replication, but are also required to promote acute infection, suggesting that PyV miRNAs function in both persistent and acute phases of infection [146].

The virally encoded SV40-miR-S1-5p contains a seed region identical to that of the human miR-423-5p and is therefore suggested to negatively regulate the expression of several miR-423-5p target genes [147]. While little is known regarding the functions of these host proteins, the Inhibitor of Growth-4 (ING-4) gene is a tumor suppressor that regulates tumor growth and angiogenesis by directly binding and modulating p53, NF-κB, and HIF-1α activity [148,149,150]. Therefore, expression of SV40-miR-S1-5p may inhibit ING-4 expression, leading to tumor cell growth, invasion, angiogenesis, and activation of the AKT and ERK1/2 signaling pathways, similarly to miR-423-5p [151]. Reciprocally, human miR-423-5p might act as a functional ortholog of the viral miRNA, causing downregulation of LTAg, limiting viral replication [140]. A recent study also revealed a high level of similarity between SV40-miR-S1-3p and human miR-1266-3p, a miRNA known to promote breast and pancreatic cancer by targeting multiple negative regulators of the STAT3 and NF-κB signaling pathways, promoting cell survival and helping to confer resistance to chemotherapy [152,153,154,155]. However, whether this viral miRNA can serve as a functional ortholog remains to be shown.

Finally, increased expression of a cellular miRNA, miR-27a, was observed during SV40 infection. Overexpression of miR-27a in SV40-infected human bronchial epithelial cells results in dysregulation of cell cycle progression and contributes to malignant transformation [156,157]. Increased expression of miR-27a has also been shown to enhance expression of proinflammatory cytokines in TLR2/4-activated macrophages via targeting IL-10, and is often associated with adverse outcomes of malignancy [158,159]. Therefore, the potent tumorigenesis induced by SV40 infection might be due to both expression of virally encoded miRNAs, miR-S1-5p and -3p, as well as the induction of an oncogenic cellular miRNA, miR-27a. Thus, like herpesviruses, polyomavirus infection induces the expression of both cellular and viral miRNAs that promote viral latency and immune evasion, and these miRNAs may also underlie the potent cellular transformation and tumorigenesis induced by polyomaviruses.

## 4. Retroviruses

**Figure 2:**
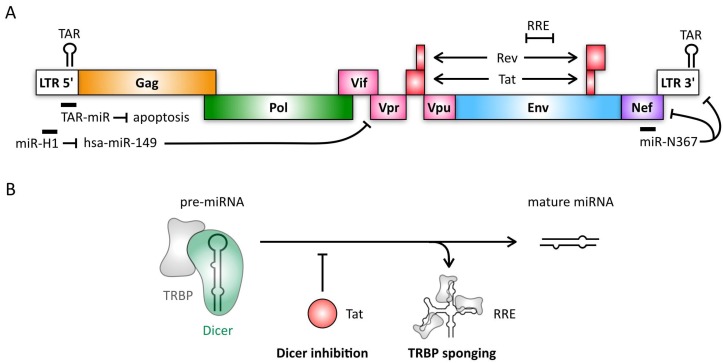
Genome organization of HIV-1 and strategies for suppression of RNA silencing. (**A**) The proviral DNA genome of HIV-1 encodes three structural genes (*gag*, *pol* and *env*) and several accessory genes (*vif*, *vpr*, *vpu*, *tat*, *rev* and *nef*) which regulate expression of viral proteins and play roles in immune evasion. The genome is flanked by long terminal repeats (LTRs), required for genome integration. Three functional miRNAs are encoded in the LTR region and Nef, which inhibit host and viral gene expression. The LTRs include the transactivation response (TAR) element stem-loop structure which can also be recognized by Dicer and TRBP and processed into TAR-miR-5p and -3p. (**B**) HIV-1 acts as a suppressor of RNA silencing. The Tat protein inhibits processing of pre-miRNAs by inhibiting Dicer activity. The Rev-response element (RRE) is a structured RNA element that resembles precursor miRNAs, able to compete with pre-miRNAs for TRBP binding.

Retroviruses are ssRNA viruses that encode a reverse transcriptase and an integrase responsible for insertion of the proviral DNA into the host genome (Figure 2A) [160,161]. Human immunodeficiency virus 1 (HIV-1) is one of the most well-studied retroviruses due to its pathogenicity in human CD4^+^ T-cells. Interestingly, potential links between miRNA expression levels and permissiveness to HIV-1 infection have been reported. Resting memory CD4^+^ T-cells, which are less permissive to infection than activated CD4^+^ T-cells, display increased levels of five cellular miRNAs that target and inhibit several HIV-1 mRNAs [162]. Accordingly, these miRNAs are highly expressed in monocytes, a cell subset that is refractory to HIV-1 infection, but that progressively becomes more susceptible upon differentiation [163]. On the other hand, several cellular miRNAs have been shown to promote HIV-1 replication and their expression levels often correlate with permissiveness to HIV-1 infection (reviewed in [164]). One such example is miR-132, which is upregulated in activated CD4^+^ T-cells when compared to resting cells [165]. MiR-132 expression promotes viral replication in Jurkat T-cells, as well as reactivation in latently infected cells. Results suggest that promotion of HIV-1 replication by miR-132 is mediated via the downregulation of a cellular transcriptional regulatory protein, MeCP2 [166,167]. However, reactivation of HIV-1 in latently infected cells was shown to be independent of MeCP2 downregulation, indicating that another mechanism is implicated in miR-132-mediated reactivation of HIV-1.

In addition to cellular miRNAs influencing HIV-1 infection, recent studies have reported the identification of HIV-1-derived miRNAs originating from coding and non-coding regions of the viral genome. Although there is controversy regarding whether these RNAs are authentic viral miRNAs based on criteria such as length and genome distribution (reviewed in [164]), several have been shown to functionally repress or promote HIV-1 replication (Figure 2A). For example, miR-N367, a negative regulatory factor (Nef)-derived miRNA, reduces HIV-1 transcription by inhibiting Nef gene expression and transcription of the long terminal repeat (LTR) region, while transactivation response element (TAR)-miR-5p and -3p both enhance infected cell survival by downregulating host genes implicated in apoptosis [168,169,170,171]. Finally, miR-H1 inhibits cellular miR-149 expression, which has been shown to downregulate viral protein R (Vpr) expression [172].

Interestingly, HIV-1 has also been demonstrated to suppress the cellular RNA silencing pathway through multiple mechanisms (Figure 2B). The HIV-1 transactivator (Tat) protein was shown to act as a suppressor of RNA silencing through an RNA-dependent interaction with Dicer [173,174]. Although this interaction functionally abrogates Dicer activity, Tat binding appears to inhibit only a subset of miRNAs and leads to phenotypic changes in the central nervous system associated with HIV-1 neuropathogenesis [175]. Therefore, it is possible that Tat binds to specific precursor miRNAs in a sequence-dependent manner, inhibiting Dicer processing of this subset of miRNAs. Additionally, a structured RNA element of the HIV-1 genome, the Rev-response element (RRE), binds to the TRBP, a central component of the Dicer complex [176,177,178]. This interaction further inhibits the RNA silencing pathway by competing with TRBP-bound RNAs [177]. Similarly to HIV-1, the primate foamy virus type 1 (PFV-1) encoded transactivator (Tas) also interferes with miRNA processing, and might function to overcome suppression of PFV-1 replication mediated by the cellular miRNA, miR-32 [179]. Thus, like the herpesviruses, retroviruses have been demonstrated to both encode viral miRNAs and modify cellular RNA silencing by inhibiting the processing of subsets of cellular miRNAs. This suppression of the cellular RNA silencing pathway may contribute to viral pathogenesis as well as cell permissibility.

## 5. Pestiviruses

**Figure 3:**
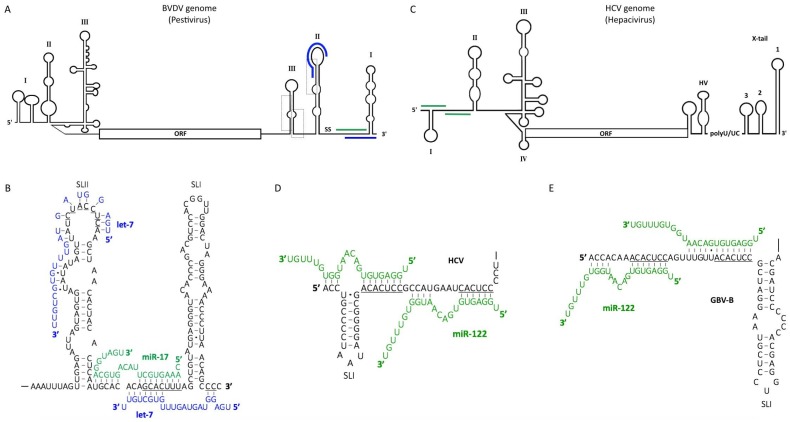
Genome organization and miRNA binding sites in the BVDV and HCV genomes. The coding regions of BVDV and HCV are flanked by 5′ and 3′ untranslated regions (UTRs) characterized by specific stem-loop (SL) structures. (**A**) The BVDV genome includes UGA box sequence elements (boxed), a single-stranded (ss) RNA region, let-7 (blue) and miR-17 (green) binding sites. (**B**) Model of the binding interactions between the BVDV 3′ UTR and miR-17 and let-7. (**C**) The HCV genome contains two miR-122 binding sites (green) and an IRES element, which includes SLII-IV in the 5′ UTR. The 3′ UTR includes a hypervariable region (HV), a polyU/UC tract of variable length and a highly conserved 3′ X-tail, comprised of SL1-3. MiR-122 binds to two tandem sites in the 5′ UTRs of (**D**) HCV and (**E**) GBV-B. The nucleotides binding to the miRNA seed regions are underlined.

Pestiviruses are small linear (+) ssRNA viruses of the *Flaviviridae* family. They commonly infect mammals, including cattle and pigs, and represent a serious threat to the food industry. Pestivirus infection can lead to diarrhea, respiratory symptoms, and reproductive dysfunctions, such as abortion [180]. In 2016, crosslinking immunoprecipitation (CLIP) studies of the Ago protein in the context of 15 different RNA virus infections revealed the functional binding of miR-17 and let-7 to the 3′ UTR of pestivirus genomic RNAs (Figure 3A,B) [181]. Canonical miR-17 and let-7 binding sites were identified in the 3′ UTR of the bovine viral diarrhea virus (BVDV) genome, specifically in the single-stranded region between SLI and SLII, and in the loop of SLII, respectively (Figure 3B) [181,182,183]. An additional non-canonical let-7 site, overlapping the miR-17 site, was supported by chimera-specific crosslinking-induced mutation site (CIMS) analysis [181]. This interaction involves extensive base pairing with the 3′ end of let-7 with only two nucleotides in the seed region (Figure 3B). Interestingly, these miR-17 and let-7 sites were both shown to be highly conserved among pestiviruses and the near-universal tropism of pestiviruses is concurrent with the ubiquitous expression of both these miRNAs across a range of tissues.

Contrasting with the canonical roles of miRNAs, binding of let-7 and miR-17 to the BVDV 3′ UTR was shown to increase both viral translation and RNA stability, with miR-17 playing a more predominant role in this regulation. Of note, Ago binding to the 3′ UTR of BVDV RNA was only observed at late time points (12–24 h post-infection) and not on replication-defective pol (-) mutant RNAs, which may suggest a role in the switch from translation to replication [181]. Mutational analyses of the non-canonical let-7 binding site revealed no effect on BVDV translation; thus, additional work is required to determine its role in the viral life cycle [181]. Binding of the miRNAs near the 3′ terminus might provide protection against 3′ exosome-mediated decay or suppress a long-range RNA–RNA interaction that is detrimental to internal ribosomal entry site (IRES) formation, similarly to models of miRNA regulation during hepatitis C virus (HCV) infection (discussed below) [184].

Interestingly, previous work suggests that the NFAR proteins bind to both the 5′ and 3′ UTRs of BVDV and help mediate genome circularization, a process promoting viral RNA translation [185]. Binding of miR-17 and let-7 at later time points during infection could therefore compete with NFAR proteins or induce conformational changes resulting in NFAR dissociation, followed by genome linearization and the switch to RNA replication. Binding of NFAR proteins to the BVDV genome involves SLII and SLIII of the 3′ UTR, with specific interactions mapped to the UGA box sequence elements [185]. Interestingly, the canonical let-7 site overlaps with the SLII UGA box element (Figure 3A,B). However, previous work demonstrated that while SLI is indispensable for pestivirus replication, deletion of either SLII or SLIII had no effect on viral translation, RNA replication, translation, packaging or particle production [186]. These results suggest that the canonical let-7 site (in SLII) is not essential for BVDV replication and that either SLII or SLIII is sufficient for recruitment of NFAR.

During viral replication, ~40% of the cellular miR-17 pool is sequestered by the BVDV genome, resulting in de-repression of many cellular miR-17 targets [187]. Conversely, let-7 sponging was <10%, likely due to the high abundance of this miRNA. However, the functional sequestration of miR-17 by BVDV did not appear to influence infection kinetics. Thus, it is unclear whether miRNA sponging by pestiviruses is simply a by-product of their dependence on a direct interaction with the viral genome, or if this mechanism also influences host gene expression in a manner that supports viral replication. The miR-17/92 cluster is highly expressed in embryonic cells and, along with let-7, might be implicated in the high rate of abortion during BVDV infection due to its key role in embryonic development [188,189,190]. The miR-17/92 cluster is also involved in lymphocyte proliferation and its overexpression can lead to cancer and autoimmune disease [191]. Since pestiviruses primarily replicate in proliferating lymphocytes, miR-17 sponging in these cells could also result in lymphocyte apoptosis or decreased proliferation. Therefore, pestivirus-induced miRNA sponging could be a mechanism of immune evasion. On the other hand, both lymphopenia and BVDV tropism could simply be a by-product of the viral dependency on miR-17, as this miRNA is abundantly expressed in proliferating lymphocytes [192]. Additional putative miRNA binding sites were also identified by Ago CLIP across the BVDV ORF, including a let-7 site in the core coding region [181]. However, the read depth of the peaks across the ORF constituted only 3–4% of the reads, calling into question the relative importance of these potential additional interactions. Thus, taken together, let-7 and miR-17 binding accounts for >50% of the miRNA binding on the BVDV genome, and >80% of the binding to the 3′ UTR. Thus, let-7 and miR-17 are important regulators of viral translation and RNA stability in pestivirus infection, and further research will help to reveal their precise mechanism(s) of regulation.

## 6. Hepaciviruses

The *hepacivirus* genus constitutes a group of hepatotropic, positive-sense, single-stranded RNA viruses of the *Flaviviridae* family that have been demonstrated to have a unique interaction with a liver-specific miRNA, miR-122. MiR-122 accounts for up to 72% of all the miRNAs found in the liver, with approximately 66,000 copies per cell, and it is highly conserved across vertebrates [193,194,195]. Although its normal role is in the regulation of cholesterol and fatty acid metabolism, it interacts with the 5′ UTR of several hepaciviruses and this interaction promotes viral RNA accumulation [196].

### 6.1. The Role of miR-122 in the Hepatitis C Virus (HCV) Life Cycle

Human miR-122 has been demonstrated to promote viral RNA accumulation in both HCV-infected cells, and in the livers of infected patients, independently of its effects on cholesterol and lipid metabolism [197,198,199]. Moreover, HCV RNA accumulation is dependent upon the miRNA biogenesis pathway, presumably due to this reliance on miR-122, as the depletion of any of the four human Ago proteins, or other key players involved in miRNA biogenesis, leads to a significant decrease in viral RNA abundance in cell culture [200,201]. MiR-122 binds to two “tandem” seed match sequences in the 5′ UTR of the HCV genome, and has additional interactions with nucleotides 1-3 and 29-31, creating a 3′ overhang at the 5′ terminus of the viral genome (Figure 3C,D) [202,203]. Stepwise mutational analyses suggest that both miR-122 binding sites are important for viral RNA accumulation and that they cooperatively support viral RNA accumulation. These analyses also indicated that nucleotides in the bulge and 3′ tail of miR-122 are important for maintaining HCV RNA abundance as mutation, truncation, or exchange of the 3′ terminal ribonucleotides of miR-122 for deoxynucleotides reduces HCV RNA accumulation. However, these nucleotides were not required for canonical miRNA activities (i.e., target cleavage and translational inhibition) [202]. These results suggest that sequences in the 3′ tail of miR-122 may mediate important interactions with viral or cellular factors involved in HCV RNA accumulation. Although the precise mechanism(s) of miR-122-mediated viral RNA accumulation have remained elusive, recent studies have suggested two major mechanisms in the viral life cycle: protection of the viral genome from 5′ decay and modification of the viral RNA structure in a manner that promotes HCV IRES-mediated translation (discussed in more detail below).

### 6.2. MiR-122 Protects the HCV Genome from Cellular Pyrophosphatase and 5′ Exonuclease Activities

The finding that miR-122 binds to the 5′ terminus of the viral RNA creating a 3′ overhang suggested a role for miR-122 in protection from nucleases or recognition by cellular sensors of RNA [202]. Recent work suggests that miR-122 binding to the 5′ terminus of the HCV genome protects the viral 5′ triphosphate moiety from recognition by cellular pyrophosphatases DOM3Z and DUSP11, and subsequent 5′ exonuclease-mediated decay [204]. Knockdown of both these cellular pyrophosphatases was shown to significantly increase viral RNA accumulation in cell culture and was also demonstrated to stabilize the HCV genome in the absence of miR-122. Moreover, knockdown of the pyrophosphatases in combination with the 5′ exonuclease (Xrn1) further increased viral RNA accumulation [204]. These observations were further confirmed by enhanced HCV replication and decreased miR-122 dependency in DUSP11 knockout cells [205]. Taken together, these results support a model whereby in the absence of the miR-122, DOM3Z and/or DUSP11 can mediate conversion of the 5′ triphosphate of the HCV genome to a monophosphate. This renders the viral genome susceptible to decay mediated by the cellular 5′ exonucleases, Xrn1 and/or Xrn2 [206,207,208,209,210]. Thus, miR-122 promotes HCV RNA stability by protecting the viral RNA from both pyrophosphatase activity and subsequent 5′ exonuclease-mediated decay.

### 6.3. MiR-122 Binding to the 5′ UTR Alters the Structure of the HCV Genome

Recent studies have also revealed that miR-122 binding to the HCV genome alters the structure of the 5′ UTR in a manner that promotes viral RNA translation. Schult *et al.* demonstrated that miR-122 binding to the viral 5′ UTR contributes to the folding of a functional IRES in an RNA chaperone-like manner [184]. *In silico* structure predictions, as well as selective 2′ hydroxyl acylation analyzed by primer extension (SHAPE) and nuclear magnetic resonance (NMR) analyses of the 5′ UTR in the absence of miR-122, identified an alternative structure for the SLII region that is more energetically favorable (SLII^alt^). This structure includes parts of SLII, preventing formation of a functional IRES element and impairing viral translation. Binding of miR-122 prevents the formation of SLII^alt^, thereby favoring SLII formation which drives the assembly of the pre-initiation complex. Indeed, polysome-profiling indicates more efficient association of the viral RNA with the 80S ribosome on either wild-type HCV RNA in the presence of miR-122, or on HCV mutants which favor SLII formation in the absence of miR-122 [211]. These observations are supported by previous studies, where *in vitro* characterization of a miR-122-sensitive double-helical switch element in the 5′ region of HCV genome indicated that a structural transition in HCV RNA conformation might impact viral translation [212]. The proposed model suggests that the IRES resides within a locked conformation, which switches to an open conformation upon interactions with miR-122. Moreover, the eukaryotic translation initiation factor 4 AII (eIF4AII), which is normally implicated in miRNA-mediated mRNA translational repression, was recently shown to interact with the HCV genome in a miR-122-dependent manner and to contribute to IRES-mediated translation [213,214]. Taken together, these results support the model whereby miR-122 binding to the 5′ UTR promotes SLII formation, leading to 80S ribosome assembly and translation initiation. MiR-122 dependency can also be further explained by the dual function of the 5′ terminal sequences in the negative strand, which represents the positive-strand promoter region. Indeed, the 3′ end of the negative strand forms an extensive set of stem-loop structures, similar to that of SLII^alt^, which are crucial for viral RNA replication [215,216]. Finally, several mutations have been identified in the HCV 5′ UTR that confer low levels of viral RNA replication in a miR-122-independent manner, and in support of this model, these mutations would be predicted to favor formation of SLII, even in the absence of miR-122 [217,218,219].

### 6.4. Dysregulation of miR-122 May Contribute to Viral Pathogenesis

Like the pestiviruses, a genome-wide miR-122 binding profile revealed functional sequestration of miR-122 during HCV infection [220]. This “sponge” effect results in de-repression of canonical miR-122 targets and deregulation of collagen production, enhanced cell proliferation and survival, and activation of hepatic stellate cells, resulting in a proinflammatory response [221,222,223]. Furthermore, miR-122 has been demonstrated to be a tumor suppressor [224,225]. Thus, in addition to promoting HCV RNA accumulation, miR-122 sequestration by the HCV genome may promote cell transformation and development of hepatocellular carcinoma.

### 6.5. MiR-122 Binding May Be a Common Strategy for Viral RNA Accumulation among Hepaciviruses

In addition to binding to the HCV genome, miR-122 binding sites have been found in the 5′ UTR of several other hepaciviruses, including GB virus B (GBV-B) (Figure 3E), non-primate hepacivirus (NPHV), several rodent hepaciviruses (RHV), and bovine hepacivirus (BovHepV) [226,227,228,229]. Although culture systems are not available for many of these novel hepaciviruses, the presence of conserved miR-122 binding sites provides hints with regard to their likely liver tissue tropism, and may suggest a conserved mechanism for viral RNA accumulation across this genus. Accordingly, both GBV-B and NPHV have been shown to be miR-122 responsive [226]. These results suggest that miR-122 binding may be a conserved mechanism for viral RNA accumulation in hepaciviruses and might help them to exploit the tolerogenic liver environment [219,230].

## 7. Conclusions

Since miRNAs are involved in all facets of cellular activities, they have major influences on viral infections and can both restrict or promote viral replication and pathogenesis. Accordingly, several viral families have evolved multiple mechanisms to take advantage of miRNAs and/or the miRNA pathway. DNA viruses, namely the herpesviruses and polyomaviruses, encode their own miRNA(s) that along with cellular miRNAs play important roles in latency maintenance, immune evasion, and tumorigenesis. Retroviruses have also been shown to encode miRNAs that modulate viral replication and pathogenesis, but are also known to encode proteins that modulate cellular miRNA processing, which is linked to viral pathogenesis and cell permissivity. In contrast, RNA viruses do not typically encode their own miRNAs; however, the pestiviruses and hepaciviruses bind to specific host miRNAs to promote viral translation, replication and/or genome stability. Of note, the ability of HCV and BVDV to use miRNAs to stabilize their genome by binding to the 5′ or 3′ UTRs, respectively, suggests that these interactions may have evolved independently [231]. Overall, these results highlight the different mechanisms by which miRNAs can influence DNA and RNA virus infection and be exploited by viruses to promote viral infection and pathogenesis. Studying these diverse interactions has provided unique insights into the canonical and non-canonical roles of miRNAs in the regulation of host and viral gene expression.

## Figures and Tables

**Table 1:** Cellular miRNAs with putative roles in herpesvirus infection.

Virus	miRNA	Targets ^1^	Predicted Roles	References
HSV-1	miR-101	ATP5B	Blocks DNA packaging and capsid maturation	[34]
		GRSF1	Attenuates viral replication	[33]
	miR-138	ICPO (v)	Inhibits lytic cycle gene expression	[36]
	miR-199a	ARHGAP21	Alters Golgi function disturbing viral envelopment	[48]
	miR-146a	Complement factor H	Immune evasion	[49]
		Arachidonic cascade	Alzheimer-type neurological changes	[49,50]
	miR-23a	IRF1	Innate immune evasion	[51,52]
	miR-649	MALT1	Innate and adaptive immune evasion	[53,54]
	miR-132	p300	Innate immune evasion	[55]
HCMV	miR-200 family	UL112 (v)	Inhibits viral reactivation	[39]
	miR-21-5p	Cdc25a	Inhibits viral replication	[56]
	miR-27b	EN2	Alters glioma cell morphology, neurological disorders	[57]
	miR-132	p300	Innate immune evasion	[55]
EBV	miR-200b, -429	ZEB1, ZEB2	Reactivation of lytic cycle, viral replication	[41,42]
	let-7	Dicer	Promotes latency	[43]
	miR-190	NR4A3	Inhibits lytic cycle	[58]
		TP53INP1	Enhances cell survival	[58]
	miR-424	SIAH1	Inhibits apoptosis	[59]
	miR-127	BLIMP1, XBP-1	Lymphomagenesis, blocks B-cell differentiation	[60]
KSHV	miR-320d, -498, -1258	RTA (v)	Inhibition of reactivation	[46,47]
	miR-132	p300	Innate immune evasion	[55]
	miR-21	Pdcd4, PTEN	Cell migration, invasion, angiogenesis	[61,62,63]
	miR-31	FAT4	Cell migration	[61,64]
	miR-146a	CXCR4	Cell migration	[65]
	miR-221/222 cluster	ETS1, ETS2	Cell migration	[64]
	miR-30b/c	DLL4	Angiogenesis	[66]

^1^ Viral targets are indicated (v).
